# Complement and *Chlamydia psittaci*: Non-Myeloid-Derived C3 Predominantly Induces Protective Adaptive Immune Responses in Mouse Lung Infection

**DOI:** 10.3389/fimmu.2021.626627

**Published:** 2021-03-04

**Authors:** Martin Kohn, Christian Lanfermann, Robert Laudeley, Silke Glage, Claudia Rheinheimer, Andreas Klos

**Affiliations:** ^1^Institute of Medical Microbiology and Hospital Epidemiology, Medical School Hannover, Hannover, Germany; ^2^Institute for Laboratory Animal Science, Medical School Hannover, Hannover, Germany

**Keywords:** complement, chlamydia, intracellular, adaptive immunity, T-cells, B-cells, C3, bone marrow chimeric mice

## Abstract

Recent advances in complement research have revolutionized our understanding of its role in immune responses. The immunomodulatory features of complement in infections by intracellular pathogens, e.g., viruses, are attracting increasing attention. Thereby, local production and activation of complement by myeloid-derived cells seem to be crucial. We could recently show that C3, a key player of the complement cascade, is required for effective defense against the intracellular bacterium *Chlamydia psittaci*. Avian zoonotic strains of this pathogen cause life-threatening pneumonia with systemic spread in humans; closely related non-avian strains are responsible for less severe diseases of domestic animals with economic loss. To clarify how far myeloid- and non-myeloid cell-derived complement contributes to immune response and resulting protection against *C. psittaci*, adoptive bone marrow transfer experiments focusing on C3 were combined with challenge experiments using a non-avian (BSL 2) strain of this intracellular bacterium. Surprisingly, our data prove that for *C. psittaci*-induced pneumonia in mice, non-myeloid-derived, circulating/systemic C3 has a leading role in protection, in particular on the development of pathogen-specific T- and B- cell responses. In contrast, myeloid-derived and most likely locally produced C3 plays only a minor, mainly fine-tuning role. The work we present here describes authentic, although less pronounced, antigen directed immune responses.

## Introduction

Complement is an essential part of the immune system. It consists of more than 50 fluid-phase and membrane-bound proteins: zymogens, mediators, regulators, and receptors. It plays a vital role in the defense against invading microorganisms. Complement is early activated in infection and functionally placed on the crossroads of innate and adaptive immunity (1). Complement factors are mainly provided by hepatocytes, but they are also produced in epithelial, endothelial, and adipose tissue and other cell types (1, 2). Local production of complement proteins by immune cells including dendritic cells (DCs), or B- and T-cells has been linked to protection (2–4). There is (mainly for humans) evidence for intracellular production of complement factors, their enzymatic activation and receptor-mediated signaling within the complement producing cells (4–8).

Complement activation occurs by three main pathways. Most complement effector functions depend on the formation of a C3 convertase and on the cleavage of the zymogen complement factor 3 (C3) into biologically active C3a and C3b. The anaphylatoxins C3a and C5a (generated by C5 cleavage further downstream in the cascade), activate leucocytes and lead to vital immunoregulatory and inflammatory responses (9). One example of a potential interrelation of complement-activating (extracellular or intracellular) pathogens and the immune system is the survival and homeostasis of resting T-cells, where the responses of human T-helper type 1 (TH_1_) cells are regulated by anaphylatoxins (6, 10–12). Moreover, these mediators promote effector functions of cytotoxic CD8^+^ T-cells (13). The anaphylatoxin receptors (C3aR, C5aR) seem to be expressed on human lymphoid cells. Yet, there are also opposing findings, for the murine system in particular, as these receptors could not be detected on lymphocytes of a non-infected anaphylatoxin receptor knock-in reporter mouse (14–17). In any case, in defense against several viruses and bacteria, APC-T-cell (co)-stimulation (14, 18) and the development of specific CD4^+^ and CD8^+^ are C3a-dependent (19–21). Moreover, complement promotes DC migration during influenza infection, as demonstrated by challenge experiments on C3^−/−^ mice (22). Newly generated C3b is highly reactive. It attaches covalently to target surfaces resulting in opsonization of pathogens and apoptotic cargo. Moreover, surface-bound iC3b and C3dg (cleavage products of C3b) lead via interaction with complement receptors to B-cell activation and antibody class switch (23–25). Additionally, C3b is interacting with CD46, a regulatory receptor expressed on human CD4^+^ T-cells and involved in TH_1_ induction processes (26). Finally, C5b bound to surfaces promotes the assembly of the membrane attack complex with lysis of accessible pathogens (1, 27, 28).

Chlamydiae are intracellular bacteria, which trigger in the form of extracellular, metabolically (almost) inactive elementary bodies (EBs) their own uptake into mucosal cells. There, they remain as dividing, metabolically active, reticulate bodies in intracellular inclusions, i.e., modified phagosomes protected against fusion with lysosomes. Thus, the unique chlamydial productive cycle resembles in some regards that of viruses. Chlamydiae re-program the host cell, which they attack and invade by translocating effector proteins via their type III secretion system. Among other effects, these chlamydial effector proteins diminish the immune response. By extrusion or cell lysis a new generation of infectious EBs is released completing the life cycle (29).

Chlamydiae cause various diseases primarily in mucosal organs in their respective hosts (30, 31). In humans, *Chlamydia pneumoniae* is responsible for (usually mild) respiratory infections (32). *Chlamydia trachomatis* is the main cause of bacteria-depending sexually-transmitted urogenital infections thereby potentially leading to infertility (33). Trachoma and blindness are caused by its serovars A–C (34). Recurrent *C. trachomatis* infections occur and immunopathology has an important role in the outcome of the disease. Development of a vaccine is faced with great challenges [for a review see (35)].

*Chlamydia psittaci* (*C.ps*.) is a zoonotic pathogen. Some strains cause intestinal, ocular and respiratory infections in birds. These avian strains are considered as BSL 3 organisms: They can be transmitted to humans who then develop severe atypical pneumonia with systemic spread of the pathogen (36) and mortality rates of up to 20% (37). In economically developed countries, few cases of human disease are reported, presumable because *C.ps*. is not included in most routine diagnostic schemes and preventive measures in bird and poultry farms can decrease frequency. Nevertheless, *C.ps*. is responsible for up to 2% of community-acquired pneumonia in some regions of Germany (38, 39).

Closely related non-avian strains, such as the *C.ps*. DC 15 isolate from an aborted calf fetus used in this study, are (for unknown reasons) usually not infective for humans (40). Thus, in some countries such as Germany, they are considered BSL-2. The frequent infection of domestic animals results in abortion, respiratory disorders, enteritis, arthritis, or economic loss due to delayed animal development (41).

### Known Links Between *Chlamydia* and Complement

In the past, there has been a misconception of complement as a strictly extracellular defense system being mostly unable to interact with obligate intracellular microorganisms. In this study, Chlamydia were selected as a model organism, because these bacteria are important zoonotic and human pathogens that reside in a unique intracellular host cell compartment during most of their life cycle, and because there are already data indicating that the complement systems plays an important role in the defense against this intracellular pathogen.

Chlamydiae activate the complement system *in vitro*: C3b binds covalently to the surface of EBs of *C. trachomatis*. Moreover, serum neutralization of *Chlamydia* is complement-dependent (42, 43). Opsonization by C3b facilitates rapid uptake of *C. trachomatis* in human monocytes (44). Furthermore, even more effectively than antibodies, complement enhances opsonophagocytosis of *C. pneumoniae* (45). Importantly, we have previously demonstrated in a lung infection mouse model that C3 and C3a with its receptor are crucial for effective protection against *C.ps*.: C3 is already activated during the first days and seems to stimulate, mainly via the C3aR, adaptive cellular immunity (46, 47). Nevertheless, our knowledge about C3-dependent immune responses and its mode of action during infections by intracellular bacteria, e.g., *Chlamydia*, is still scarce. From a phylogenetic point of view, the well-being of the main host of obligate intracellular pathogens, such as chlamydiae, is a pre-requisite for their survival. Consequently, such organisms have to comply with a less aggressive infection strategy. Hence, it is not surprising that chlamydial diseases tend to be subacute, long-lasting, or even chronic in their main host (31). Due to the relatively long duration of these infections, interactions of different components of host defense can be investigated in corresponding animal models over prolonged periods. This is, in particular, helpful for interactions concerning the development of specific immune responses and the resulting protection. Complement is an important immunomodulatory player during infection. It has been assumed that local production of complement by myeloid-derived cells is crucial in this context (9, 48, 49).

On this background, the present study elucidates the role of myeloid cell-derived vs. non-myeloid cell-derived complement, for the development of the adaptive cellular and humoral immune response and effective protection against the intracellular bacterium *C.ps*. in mouse lung infection. For that purpose, challenge experiments with *C.ps*. were combined with adoptive bone marrow transfer using C3^+/+^ and C3^−/−^ mice.

## Materials and Methods

### Chlamydial Culture

The DC 15 strain of *C.ps*. isolated from bovine abortion (50) was kindly provided by K. Sachse (NCBI GenBank accession: CP002806.1) and propagated in BHK-21 cells as described elsewhere (47). The German government and corresponding authorities regard “non-avian *C.ps*. strains” BSL 2 (http://www.zkbs-online.de). Inclusion-forming units (IFU) were assessed by titration using HeLa-T cells (51). Mock infected controls were prepared identically, but without *Chlamydia*, and diluted to the same extent as harvested infected cells. All preparations were Mycoplasma-free tested by PCR.

### Animal Experiments and Mouse Strains

All animal experiments were approved by the Lower Saxony state government and corresponding authorities of the Lower Saxony State Office for Consumer Protection and Food Safety (LAVES) (permit: 33.12-42502-04-18/2751). Experiments were performed in accordance with the law of animal welfare used for experiments (TierSchVersV), with the German regulations of GV-SOLAS for the protection of animal life and FELASA. In this work, the following strains were used: C3^−/−^ [B6.129S4-C3tm1Crr/J– (52)] C3^+/−^, Wildtyp (WT) CD45.2 C57BL/6J (JAX-ID 002014), and WT CD45.1 C57BL/6J (JAX-ID 002014) mice. All C3^−/−^ mice were backcrossed on C57BL/6J background for at least 6 additional generations.

### *Chlamydia psittaci* Mouse Lung Infection

Intranasal infection of male mice was performed under anesthesia using 100 mg/kg BW Anesketin (Dechra, 08714225156146), 4 mg/kg Rompun (Bayer, PZN-01320422) in 0.9% NaCl as described elsewhere (53). Depending on the setting, mice were infected with an IFU of 750, or 1.7 × 10^3^ per mouse, as indicated. For Mock infection mice were treated identically, applying the same volume of mock material obtained from cultured non-infected BHK-21 cells (see above in “Chlamydial culture”). All infected animals were observed daily assessing clinical score (for details see table in Supplementary Material) and body weight.

### Lung Histopathology

Classification and scoring (for details see table in Supplementary Material) of lung histopathology by light microscopy on Hematoxylin and Eosin (Merck) stained deparaffinized, “blinded” lung sections was performed as previously described (47).

### Cryo-Conservation of Lung Tissue and Preparation of Single Cells Suspensions From the Lung Draining Lymph Nodes

Organs from infected and mock treated dissected mice and lung homogenate were cryo-conserved as previously described (47). To obtain single cell suspensions, lymph nodes (LN) were collected in PBS+10% FCS passed through a 40 μM nylon mesh (BD, 352340) using a syringe stamp and flushed two times with 10 ml of cold PBS. If necessary, red blood cell lysis was performed using ACK lysis buffer (155 mM NH_4_Cl, 10 mM KHCO_3_, 0.1 mM EDTA).

### Generation of Bone Marrow (BM) Chimeric Mice

Eight- to nine-week old male C57BL6/J CD45.1-WT and CD45.2-C3^−/−^ mice were total-body-irradiated with 11Gy using the BioBeam GM 2000 (Gamma-Service Medical GmbH). Six hours post-irradiation 2 × 10^7^ bone marrow cells from either CD45.2-C3^−/−^ or CD45.1-/CD45.2-WT mice were adoptively transferred intravenously to each irradiated animal. During BM engraftment (in the following 5 weeks), 0.05 mg/ml ciprofloxacin was added to the drinking water. Number of myeloid cells in blood and reconstitution efficiency was controlled 6 weeks post irradiation by flow cytometry using APC anti-mouse CD45.1 (BioLegend, 110714) and FITC anti-mouse CD45.2 (BioLegend, 109806). Only mice exceeding BM exchange >95% were used for infection experiments 8 weeks post-irradiation. In addition, comparable leucocyte cell counts were achieved in the four different combinations of chimeric mice (data not shown).

### Mouse C3, and Anti-*Chlamydia psittaci*-Specific Mouse IgM, IgG, and IgA ELISAs

For C3 and the anti-*C.ps*.-specific Ig ELISAs, microtitre plates were coated either with C3 capture antibody (Hycult, HM1065 1 μg/ml), or lysate of *C.ps*. (0.11 μg protein/well, corresponding to 2.48*10^5^ IFU /well, inactivated at 80°C 30 min and sonified for 15 min), or *C.ps*.-free mock cell-homogenate (0.11 μg protein/well) as antigen—and blocked with 1% BSA/5% sucrose. C3 and anti-chlamydial antibodies were determined in EDTA-plasma (1 mM EDTA PBS, pH of 7.4; stored at −80°C) collected from the tail vein during the ongoing experiment, or from heart puncture (47) at the end of the observation period, respectively. Samples diluted in 20 mM Tris, 150 mM NaCl, 0.1% BSA and 0.05% Tween-20 were incubated for 1.5 h at 37°C. Detection antibodies were added for 45 min at 37°C (C3 ELISA: 0.5 μg/ml biotin rat anti-mouse C3a, Hycult, HM1072-B, followed by 10 μg/ml streptavidin-conjugated HRP, Jackson Immuno Research, 016-030-084; IgM ELISA: 1:1000 HRP rat anti-mouse-IgM, BD Biosciences, 550588; IgG ELISA: 1:1000 HRP rat anti-mouse-IgG, Dianova, 115-036-062; IgA ELISA: 1:1000 HRP rat anti-mouse-IgA, antibodies-online, ABIN135043). Substrate buffer (90 mM Na-acetate, 90 mM citric acid, 100 μg/ml TMB, 0.0045% H_2_O_2_) was added for 20 min in the dark, stopping the enzymatic reaction by 1M H_2_SO_4_. The amount of C3 or the anti-*C.ps*. Ig was calculated from the absorbance (450/540 nm). Calculation was performed in Graphpad Prism applying the inverse function of the four parametric logistic equation using standard curves either consisting of pooled C57-BL6/J mouse plasma (1:3 in PBS 10 mM EDTA pH 7.4) or of protein Ig-standards provided by the manufacturer.

### SDS-PAGE and Western Blot for Mouse C3 Detection

Blood plasma diluted in PBS, 10 mM EDTA, pH7.4 (see above) or lung homogenate with 1 × proteinase inhibitor (Roche, 11836153001) were mixed to an equal volume with 5xLaemmli buffer without reducing agents. Samples were not boiled prior to loading to minimize dissociation of C3 into its alpha and beta chains. SDS-PAGE was performed on 10% resolving gel. After transfer to Whatman^®^ Protran^®^ nitrocellulose membrane (GE Healthcare), mouse C3 was detected using goat anti-mouse C3 antiserum (GENETEX, GTX72994). Protein quantification was assessed using ImageJ v1.52e software (Wayne Rasband National Institute of Health). Band intensity was calculated with reference to the same internal C3 mouse pool plasma standard from healthy WT mice used in the C3-ELISA. Three standard dilutions were included on each gel/Western blot: 1:1, 1:4, and 1:20. The limit of detection (LOD, indicated in the graphs by a dotted horizontal line) caused by unspecific background was defined as the signal intensity obtained with the highest of several C3^−/−^➔C3^−/−^ controls of Mock-infected or *C.ps*.-infected mice, respectively. The background was not subtracted in the graphs to obtain better insight in the results, and to get even in negative controls calculated very low, hypothetical signal-intensities. That permits the performed statistical comparison of samples containing low levels of C3 (or even none) with those containing higher concentrations.

### TNF-α, IFN-γ, and Myeloperoxidase (MPO) ELISA

These ELISAs were performed on mouse lung homogenate (in similar fashion) according to the manufacturer's protocol (MPO: MPO, Mouse, ELISA kit, Hycult Biotech, HK210-02; IFN-γ: ELISA MAX™ Deluxe Set Mouse IFN-γ, BioLegend, 430804; TFN-α: ELISA MAX™ Deluxe Set Mouse TFN-α, BioLegend, 430904).

### Flow Cytometric Determination of Bacterial Load in Lung Homogenate

1.6 × 10^4^ HeLa-T cells were seeded into a 96 well and cultured in MEM medium 24 h prior to infection. On the next day, cells were washed once with PBS and medium was changed to RPMI-1640 containing 10% FCS. Cryopreserved lung homogenates were thawed 15 min on ice, samples were vortexed for 3 min and centrifuged for 15 min, 500×g, 4°C. The collected sample supernatant was serial diluted in RPMI-1640, 10% FCS, and used to infect HeLa-T cells. In addition, a dilution series of *Chlamydia* stock preparation was used as internal standard to calculate bacterial content in lung homogenate samples. Next, cells were spinoculated with diluted supernatant or *C.ps*. stock dilution for 1 h, 2,000×g, 35°C. 20 h after infection, cells were washed with PBS and trypsinated with Trypsin/EDTA for 5 min. RPMI-1640 10% FCS was added to stop enzymatic digestion and cells were transferred to a v-shaped 96 well-plate suitable for staining procedure. Next, cells were spinned down for 5 min, 1,200×g, 4°C, supernatant was removed and cells were resuspended in PBS containing 2% formaldehyde to fix cells for intracellular staining. After fixation cells were centrifuged (5 min, 1,200×g, 4°C), supernatant was removed and washed with PBA-S (PBS supplemented with 0.25% BSA, 0.02% sodium azide, 2 mM EDTA, 0.5% Saponin) to permeabilize cells. Staining of intracellular chlamydiae and cells was performed for 40 min in PBA-S at 4°C using Pathfinder^®^ containing Fluorescein-conjugated murine monoclonal antibody to chlamydial LPS and 0.1% Evans Blue (0.54 μl per well; Bio-Rad). After staining cells were washed with PBA-S, centrifuged (5 min 1,200×g, 4°C) and resuspended in PBA for flow cytometric data acquisition. Flow cytometry acquisition was performed with a MACSQUANT Analyzer 10 (Miltenyi Biotec) and data analyzed with FlowJo v.10.4.2 (Becton, Dickinson and Company (BD). The limit of detection (LOD, indicated in the graphs by a dotted horizontal line) caused by unspecific background fluorescence was based on the results obtained with the highest of several non-infected Mock controls. Background fluorescence (caused by artifacts or cell debris) was not subtracted in order to get even in negative controls calculated very low, hypothetical chlamydial counts. This practice permitted statistical comparison of samples containing only few chlamydia (or even none) with those containing higher amounts.

### Immune Phenotyping of the Cells in the Mouse Lung Draining Lymph Nodes

Single cell suspensions from lung dLN (as described above) were used to perfom immune cell phenotyping using the following antibodies: Pacific Blue™ anti-mouse CD3 (BioLegend, 100213), FITC anti-mouse CD19 (BioLegend, 115506), FITC anti-mouse CD45.2 (BioLegend, 109806), VioBright-FITC anti-mouse CD44 130-120-287, PE anti-mouse CD45.1 (BioLegend, 110708), PE anti-mouse CD279 (PD-1) (Miltenyi Biotec,130-111-953), PerCP/Cy5.5 anti-mouse CD8a (BioLegend, 100734), PE/Cy7 anti-mouse CD4 (BioLegend, 116016), PE/Cy7 anti-mouse CD62L antibody (BioLegend, 104417), APC anti-mouse CD4 (BioLegend, 100516), APC anti-mouse CD45.1 (BioLegend, 110714), APC anti-mouse CD3ε antibody (BioLegend, 152306). For life and dead staining of cells, either DAPI (BioLegend, 422801) or zombie aqua^TM^ (BioLegend, 423102) were used. In brief, cells were washed with PBS, followed by life/dead staining (15 min on ice). Cells were washed with PBS and resuspended in 5 μg/ml anti-mouse CD16 and CD32 (BioLegend, 101301) in PBA for 20 min (PBS containing 0,25% BSA, 0,02% sodium azide, 2 mM EDTA). After Fc Block antibody and washing with PBS, immune-cell staining was performed in PBA containing fluorochrome conjugated antibodies (30 min on ice). After several washing steps, cells were fixed in PBS containing 2% formaldehyde (20 min on ice), washed once more and resuspended in PBA ready for acquisition. For most antibodies, the corresponding isotype controls were used. Flow cytometry acquisition was performed as described above.

### APC-T-Cell Re-Stimulation and Quantification of *C. psittaci*-Specific Cellular Response

BM was isolated from the femur or tibia of 8–12-week-old mice. BM cells were resuspended in BM DC media (RPMI-1640, 10% FCS, 100 U/ml Penicillin/Streptomycin, 50 μM β-Mercaptoethanol) containing 10 ng/ml GMCSF. Fresh BM DC media supplemented with 5 ng/ml GMCSF was partially replaced every 4 and 6 days. After 7 days of cultivation, cells were removed using a cell scraper, washed with cold PBS and placed on ice. Cell number was determined by flow-cytometry and 1.25 × 10^5^ BM derived dendritic cells (BM DCs) were seeded into each well of a 24-well in (-)BM DC media (without antibiotics). Subsequently, cells were infected with *C.ps*. (multiplicity of infection = 1) or mock treated in (-)BM DC media with of 5 ng/ml GM-CSF and 10IU of IL-4 (Miltenyi Biotec, 130-097-757). 24 h later, lymphocytes from the dLN of infected mice were isolated and co-cultured to a 1:8 ratio with mock or *C.ps*. infected BM DCs in (-)BM DC-media with final concentrations of 5 ng/ml GM-CSF and 120IU IL-2 (Miltenyi Biotec, 130-097-743) for 16 h. During the last 2 h of co-culture, cells were treated with 5 μg/ml of Brefeldin A solution (BioLegend, 420601) to block Golgi-associated protein transport. Subsequently, cells were harvested and placed on ice. Immune cell phenotyping was performed as described above after fixation of cells. In addition, intracellular cytokine staining of the lymphocytes was performed with IFN-γ (Miltenyi Biotec, 130-102-388) in PBA-S for 40 min on ice after permeabilization of cells for 20 min with 0.25% BSA, 0.2% sodium azide 2 mM EDTA, 0.5% Saponin in PBS. The chlamydia specific T-cell response was evaluated according to percentile of IFN-γ positive cells on either mock or *C.ps*. re-stimulated BM DCs.

## Calculations and Statistics

GraphPad Prism V8.0.1 (GraphPad Software Inc.) was used for graphical display and statistical evaluation. Usually, infected and mock-treated animals were compared first using an unpaired *t*-test. If significant *C.ps*.-dependent differences were found, further statistical analyses were applied. Normal distribution of parametric data was checked for each data set. In the majority of cases logarithmic transformation of data was performed in order to accomplish Gaussian distribution. Parametric data were analyzed either by unpaired *t*-test (2 groups, 1 condition), One-way (2 ≤ groups, 1 parameter) or Two-way ANOVA (2 ≤ groups, 2 ≤ parameter) followed by Bonferroni multiple comparison test. In general mean ± SD (standard deviation) was used to represent parametric data. In some cases, if the sample size exceeded *n* ≥ 6, mean ±SEM (standard error of mean) was used. Non-parametric data were shown as mean ± IQR (interquartile range) and analyzed by Kruskal-Wallis test followed by Dunn's multiple comparison post-test. Details and group size of each experiment is depicted within or under each corresponding figure. Survival of mice was analyzed by Mantel-Cox log-rank test. Statistical significance was displayed and presented according to the calculated *p*-value: ns *p* > 0.1234; *p ≤ 0.0332; ^**^p ≤ 0.0021; ^***^p ≤ 0.0002; ^****^p ≤ 0.0001.

## Results

### Both, Non-Myeloid and Myeloid-Derived Complement Are Engaged in the Defense Against *C. psittaci* in Mouse Lung Infection

**Figure 1:**
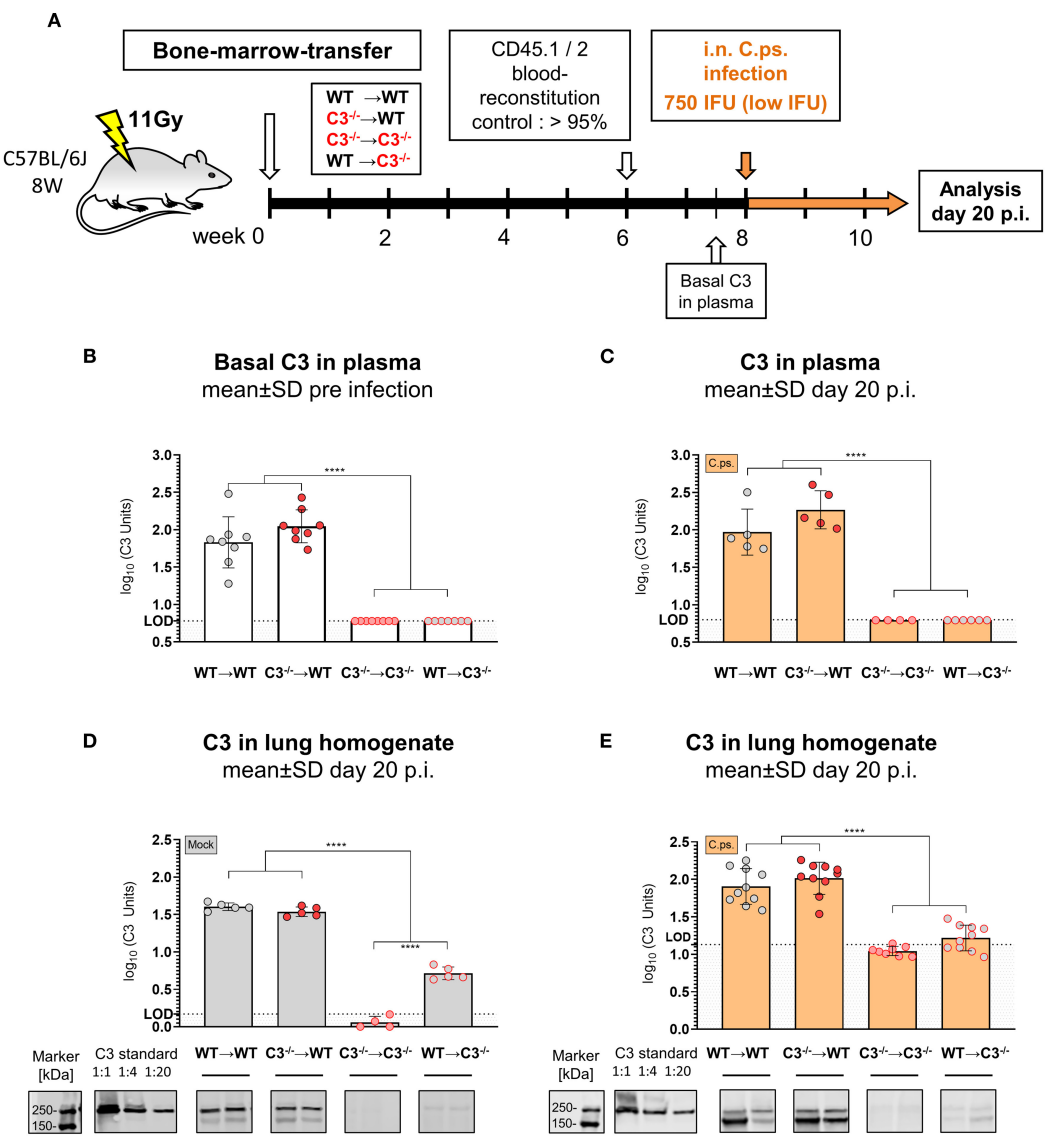
C3 levels in plasma and lung homogenate of BM chimeric mice before and on day 20 of *Chlamydia psittaci* mouse lung infection. C3^+/+^ and C3^−/−^ mice were irradiated and adoptive transfer of bone-marrow (BM) from C3^+/+^ and C3^−/−^ mice was performed to generate the 4 different possible combinations of chimeric mice: WT➔WT, C3^−/−^➔WT, C3^−/−^➔C3^−/−^, and WT➔C3^−/−^ animals (i.e. donor BM in recipients). Six weeks post-irradiation, reconstitution of the expected altered BM of >95% was confirmed in peripheral blood using CD45.1 and 2 congenic markers. Eight weeks post-irradiation, the chimeric mice were infected with 750 IFU of *C.ps*. **(A)**. C3 in plasma was determined by ELISA using pooled EDTA plasma from WT mice, defined as 100U, as standard **(B,C)**. For technical reasons, C3 in lung homogenate was determined by Western blot **(D,E)**. The blots of two mice of each group are depicted as examples. The calculated band intensities of all mice of each group were normalized to serial diluted pooled C3-plasma. The displayed results were obtained combining 4 independent experiments. The bars representing results obtained on non-infected control mice are filled in gray; the bars obtained with *C.ps*.-infected mice are filled in orange. Statistical analysis was performed by One-way ANOVA followed by Bonferroni post-test. Statistical significance according to the calculated *p*-value: *p ≤ 0.0332; **p ≤ 0.0021; ***p ≤ 0.0002; ****p ≤ 0.0001. Illustrations were partially created using templates from www.motifolio.com. LOD: limit of detection.

In order to elucidate the contribution of myeloid and non-myeloid-derived complement in defense against intracellular chlamydiae, C57BL/6J WT (C3^+/+^) and C3^−/−^ mice were irradiated and adoptive BM transfer was performed to generate chimeric mice for *C.ps*. challenge experiments (Figure 1A). Titration experiments had shown that mice were slightly more susceptible to chlamydia after irradiation and adoptive BM transfer (data not shown). Based on the preliminary testing, WT➔WT, C3^−/−^➔WT, C3^−/−^➔C3^−/−^, and WT➔C3^−/−^ mice (i.e., donor BM in recipients) were i.n., infected with 750 IFU of *C.ps*. (Figure 1A). This low IFU infection permitted prolonged survival and thus, detailed analysis of tissue, immune cells, and pathogen specific immune responses of all animals 20 days after infection (*p.i*.).

### Levels of C3 in Chimeric Mice

The levels of C3 (determined by ELISA) were similarly high in EDTA-plasma of both chimeric WT recipients before (Figure 1B) as well as 20 days after *C.ps*. infection and recovery (Figure 1C). In contrast, C3 was neither detectable in blood of healthy C3^−/−^➔C3^−/−^ nor of WT➔C3^−/−^ mice (Figure 1B), and it remained below the limit of detection 20 days *p.i*. (Figure 1C). Yet, it should be noted that the detection limit (L.O.D.) of our ELISA is (according to the determination of dilutions of pool plasma—data not shown) equivalent to ~5–7% of the level of C3 of standard pool plasma from healthy WT C57BL/6J mice. From that one can draw the conclusion that approx. ≥95% of the circulating C3 in WT➔WT BM chimeric mice during steady state and during *C.ps*. infection is derived from non-myeloid cells, most likely, primarily of the liver—and up to 5% might be produced and secreted by non-myeloid cells. This modest interpretation of our results is in accordance with reports of others obtained after BM and liver transplantation (54–56).

Lung homogenate was not suited for determination of C3 in the mouse C3-ELISA because the analysis of this material led even in C3^−/−^ mice (in contrast to EDTA-plasma from the same animals) to a high, most likely non-specific background. Yet, by Western blot a small amount of C3, equivalent to <1:20 of diluted WT pool plasma used as “C3 standard” also in the ELISA, seems to be present in the lung homogenate of Mock*-*infected WT➔C3^−/−^ compared to C3^−/−^➔C3^−/−^ mice as determined on day 20 (Figure 1D). The background signal in lung homogenate of C3^−/−^➔C3^−/−^ mice was higher after infection (Figure 1E). Most likely, for that reason, the difference between the lung homogenates of WT➔C3^−/−^ and C3^−/−^➔C3^−/−^ mice remained then only a trend.

### Chlamydial Infection of the Chimeric Mice

**Figure 2:**
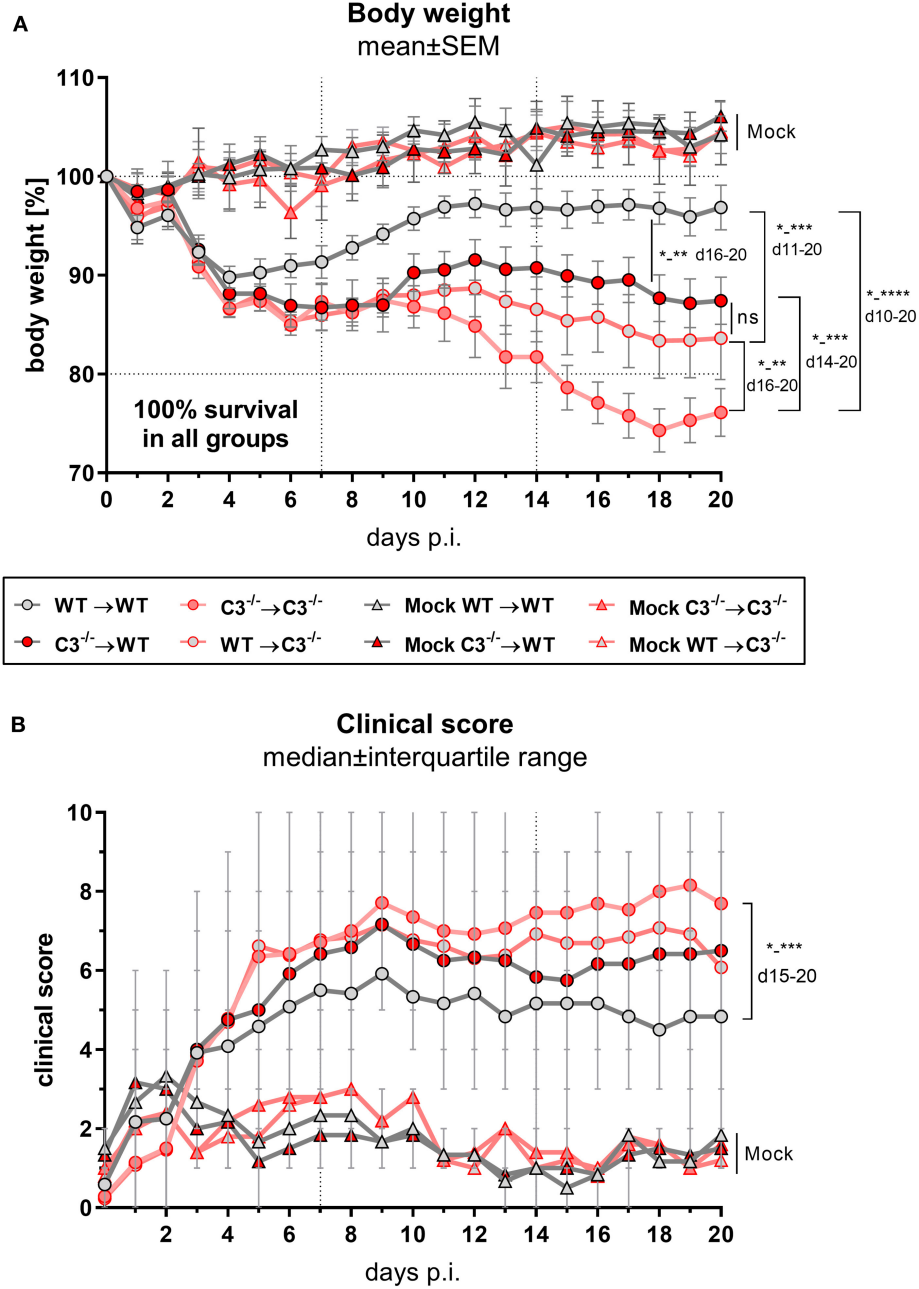
Body weight and clinical score of BM chimeric mice during *C. psittaci* mouse lung infection indicate that both, myeloid and, to a greater extend, non-myeloid cell-derived C3 is required for maximal protection. BM chimeric mice (see also legend of Figure 1A) were infected with 750 IFU of *C.ps*. Body weight **(A)** and clinical score **(B)** were assessed daily until day 20 *p.i*.. The depicted results were obtained combining 4 independent, identical experiments. Statistical analysis was performed by Two-way ANOVA **(A)** followed by Bonferroni post-test, or by Kruskal-Wallis test followed by Dunn's multiple comparison post-test **(B)**. Statistical significance according to the calculated *p*-value: *p ≤ 0.0332; **p ≤ 0.0021; ***p ≤ 0.0002; ****p ≤ 0.0001.

**Figure 3:**
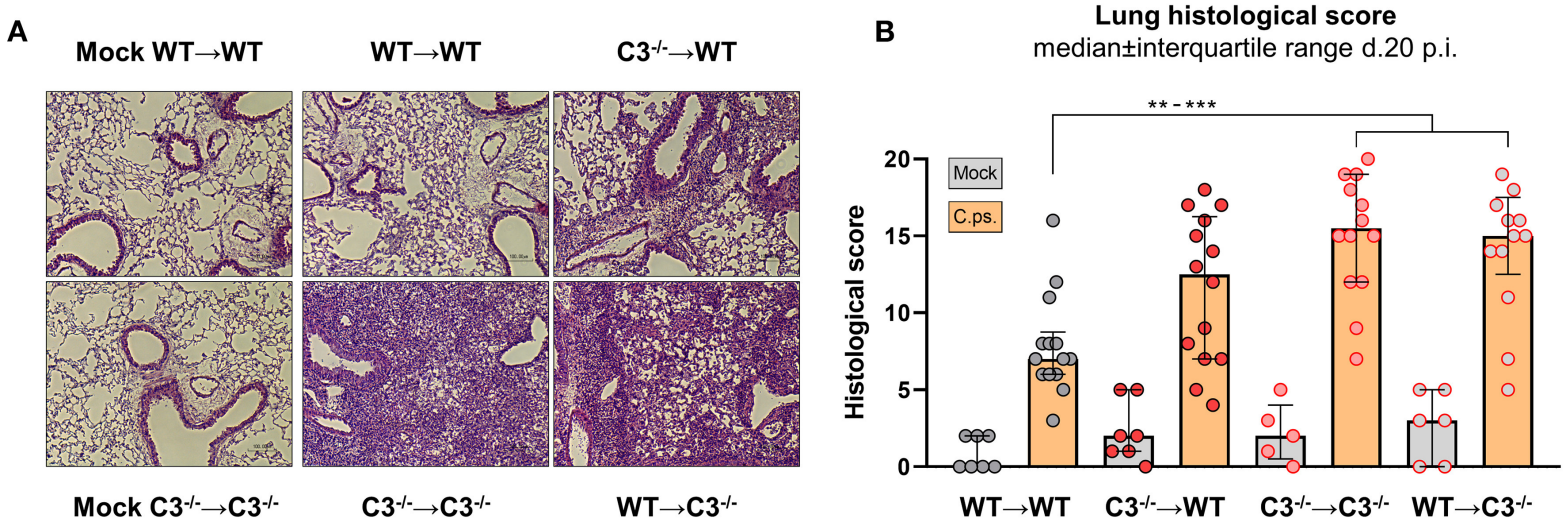
Lung histology including score of BM chimeric mice on day 20 of *C. psittaci* infection indicate that in particular non-myeloid cell-derived C3 is required for protection. BM chimeric mice (see also legend of Figure 1A) were infected with 750 IFU of *C.ps*., and analyzed 20 days *p.i*.. Lung histology was assessed: One representative histological section of each group of infected mice and of two mock-infected controls is depicted **(A)**, as well as the histological inflammatory score of H&E stained lung sections determined by a blinded observer **(B)**. The displayed results were obtained combining 4 independent, identical experiments. The bars representing results obtained on mock- or non-infected control mice are filled in gray; the bars obtained with *C.ps*.-infected mice are filled in orange. Statistical analysis was performed by One-way ANOVA followed by Bonferroni post-test **(B)**. Statistical significance according to the calculated *p*-value: *p ≤ 0.0332; **p ≤ 0.0021; ***p ≤ 0.0002; ****p ≤ 0.0001.

**Figure 4:**
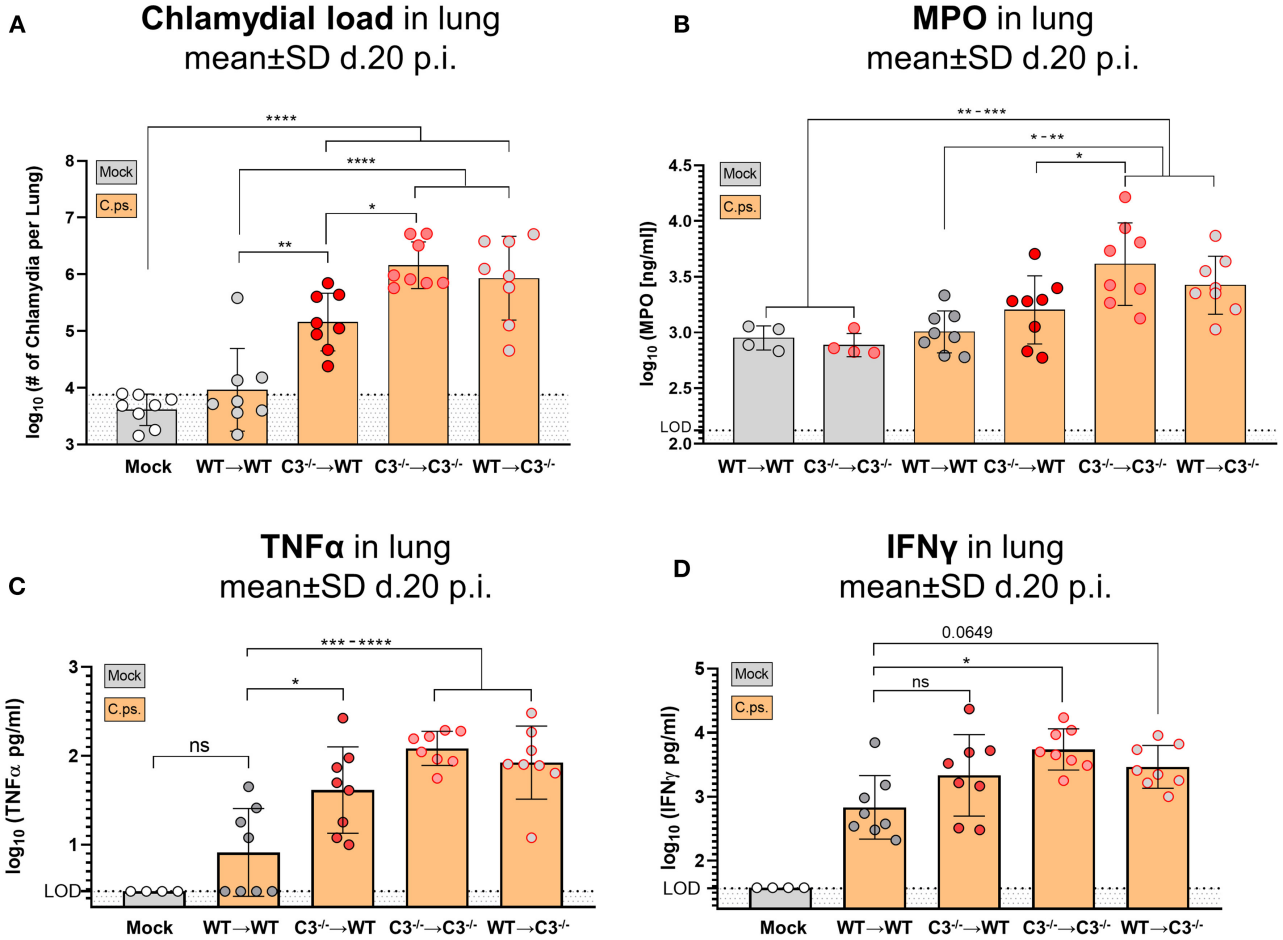
Chlamydial load, and granulocyte marker MPO, TNF-α and IFN-γ in lung homogenate of BM chimeric mice on day 20 of *C. psittaci* infection indicate that non-myeloid cell-derived C3 is required for protection. The burden of infectious *Chlamydia* **(A)**, the granulocyte marker MPO **(B)**, as well as the key cytokines TNF-α **(C)** and IFN-γ **(D)** were determined in lung homogenate of BM chimeric mice (see also legend of Figure 1A) obtained on day 20 *p.i*. with 750 IFU of *C.ps*. Background fluorescence caused by artifacts or cell debris was not subtracted in the flow cytometric determination of *Chlamydia* **(A)**. The displayed results were obtained combining 4 independent, identical experiments. The bars depicting results obtained on mock- or none-infected control mice are filled in gray; the bars obtained with *C.ps*.-infected mice are filled in orange. Statistical analysis was performed by One-way ANOVA followed by Bonferroni post-test. Statistical significance according to the calculated *p*-value: *p ≤ 0.0332; **p ≤ 0.0021; ***p ≤ 0.0002; ****p ≤ 0.0001. LOD, limit of detection.

The four different groups of infected chimeric animals showed a similar loss of body weight (Figure 2A) and increased clinical scores 3–4 days after *C.ps*. infection (Figure 2B). In contrast, mock-infected animals of the four groups showed only a slightly elevated score without any relevant clinical symptoms (Figures 2A,B). With respect to these parameters, the illness of the infected WT➔WT mice reached its maximum after 4–7 days. According to the body weight, these animals recovered partially to a substantially improved health status until day 10–12. Nevertheless, compared to mock-infected animals their overall appearance (i.e., clinical score in Figure 2B) and the lung histology (Figure 3A + histological score in Figure 3B) were still impaired to some extent until day 20, suggesting even in WT➔WT mice surprisingly long-term effects of chlamydial lung-infection on inflammation. These lesions were mainly due to slightly increased number of inflammatory cells and remaining edema (see also heat map of the different categories within our histological score and the table in Supplementary Material). Yet, at this time point, the amount of infectious *C.ps*. in the lung was already below the limit of detection in most of the WT➔WT animals (Figure 4A).

Compared to WT➔WT mice, the course of the illness of chimeric C3^−/−^➔C3^−/−^ animals appeared to be worse already after 6–10 days: Beginning on day 10 or 15, respectively, the mice without any sources of C3, deteriorated more than the other groups; the difference in body weight reached significance from day 16 on (Figures 2A,B). This was reflected by a higher histological score (Figure 3B) and an ~100-fold higher bacterial load, as well as higher levels of the key cytokines IFN-γ and TNF-α in lung homogenate on day 20 *p.i*. (Figures 4A,C,D).

Intriguingly, both mixed chimeras, C3^−/−^➔WT as well as WT➔C3^−/−^, showed a relatively similar, intermediate phenotype. Both lost more weight than infected WT➔WT but less than C3^−/−^➔C3^−/−^ animals (Figure 2A). This strongly suggests that C3 derived from myeloid- and from non-myeloid sources is participating in defense against chlamydial lung infection.

The amount of the granulocyte marker MPO in the infected lung was smaller in WT➔WT and C3^−/−^➔WT mice as compared to C3^−/−^➔C3^−/−^ animals suggesting a shorter non-specific phase of inflammation and faster recovery in the presence of non-myeloid cell-derived C3 (Figure 4B). As indicated by the elevated MPO level in WT➔WT mice on day 20, even in presence of optimal levels of C3 and despite the absence of detectable viable bacteria, granulocyte driven inflammation seemed to be relatively persistent.

Intriguingly, the mixed WT recipient was frequently closer to the WT➔WT phenotype than the mixed C3^−/−^ recipient: For instance, only in C3^−/−^➔WT, but not in WT➔C3^−/−^ mice, the amount of chlamydia in the lung was lower than in C3^−/−^➔C3^−/−^ animals (Figure 4A). These results indicate already a higher relevance of non-myeloid cell-derived C3.

### Non-Myeloid Cell-Derived C3 Is Predominantly Required to Induce Protection and to Promote the Induction of *C. psittaci*-Specific T- and B-Cell Responses

**Figure 5:**
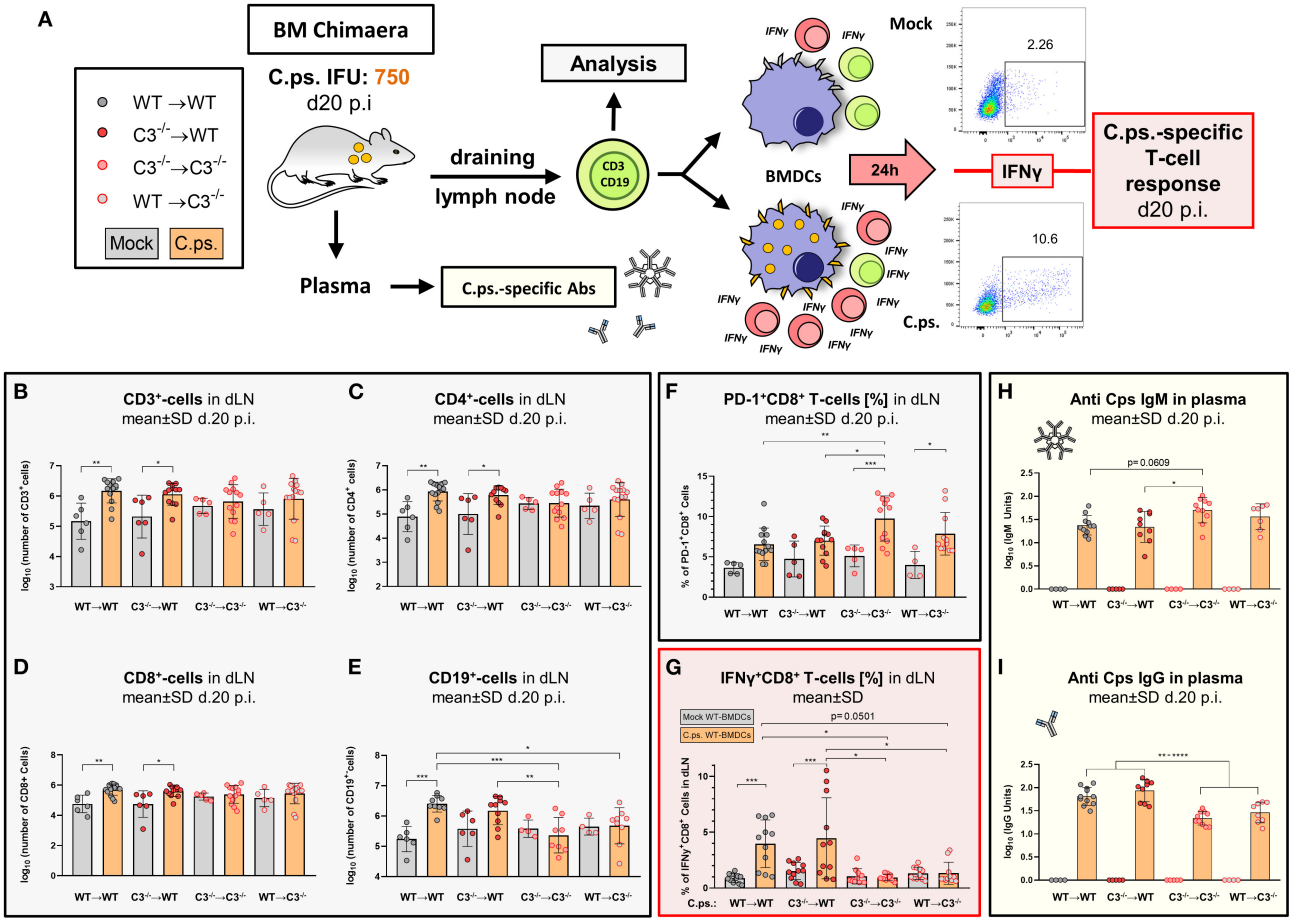
*Chlamydia psittaci*-specific B- and T-cell responses depend on the presence of non-myeloid cell-derived C3. Twenty days after infection with 750 IFU of *C.ps*., the BM chimeric mice (see also legend of Figure 1A) were sacrificed. Lung dLN were isolated to directly analyze B- and T-lymphocytes by flow cytometry, and to characterize *C.ps*.-specific T-cell responses after 16 h of re-stimulation with *C.ps*. infected BM derived WT-DCs. The *C.ps*.-specific antibody response was determined in EDTA-plasma **(A)**. The number of different lymphocyte subsets in dLN **(B–E)**, the expression of the exhaustion marker PD-1 on CD8^+^ T-cells **(F)**, as well as the percentage of IFN-γ^+^CD8^+^-T-cells after re-stimulation (G) are depicted. *C.ps*.-specific IgM, IgG in plasma was determined by ELISA **(H,I)**. The displayed results were obtained combining 4 independent identical experiments. The bars representing results obtained on mock- or non-infected control mice are filled in gray; the bars obtained with *C.ps*.-infected mice are filled in orange. Statistical analysis was performed by One-Way ANOVA followed by Bonferroni post-test. Statistical significance according to the calculated *p*-value: *p ≤ 0.0332; **p ≤ 0.0021; ***p ≤ 0.0002; ****p ≤ 0.0001. Illustrations were partially created using templates from www.motifolio.com.

To elucidate how far myeloid- and non-myeloid cell-derived C3 is required to establish T- and B-cell responses, lymphocytes in the lung draining lymph nodes and chlamydia-specific antibodies in blood of the chimeric mice were analyzed 20 days *p.i*. (Figure 5A).

Lack of non-myeloid C3 impeded the *C.ps*.-dependent increase in the number of CD3^+^, CD4^+^, and CD8^+^ T-cells in both chimeric recipient mice (Figures 5B–D), although there was no difference comparing the absolute numbers of these cells in the four different combinations of BM chimeric mice after infection.

**Figure 6:**
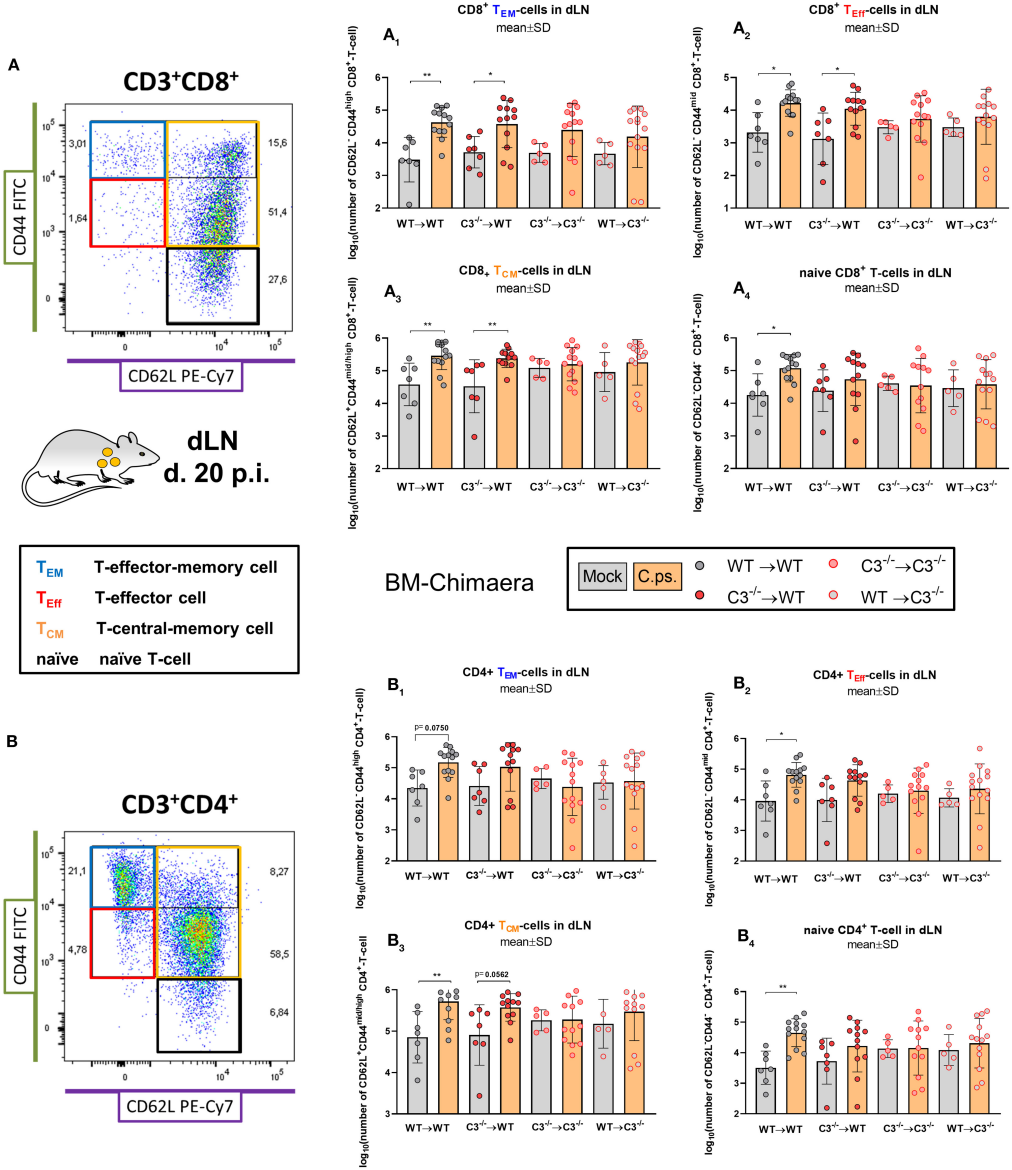
Formation of CD8^+^ and CD4^+^ effector T-cells determined on day 20 *p.i***.** BM chimeric mice (see also legend of Figure 1A) were infected with 750 IFU of *C.ps*.. On day 20 *p.i*., mice were sacrificed and dLN were isolated for flow cytometric analysis of T-lymphocytes. The number of CD8^+^ and CD4^+^ naïve and effector T-cells in the dLN (A1 to 4, or B1 to 4, respectively). Statistical analysis was performed by One-way ANOVA followed by Bonferroni post-test. Data were combined from 4 independent identically performed experiments. Statistical significance according to the calculated *p*-value: *p ≤ 0.0332; **p ≤ 0.0021; ***p ≤ 0.0002; ****p ≤ 0.0001. Illustrations were partially created using templates from www.motifolio.com. TEM, T-effector memory cell; T_Eff_, T-effector cell; TCM, T-central memory cell.

In addition, the exhaustion marker programmed cell death protein-1 (PD-1) was upregulated on CD8^+^ T-cells of both C3^−/−^ recipients (Figure 5F). Furthermore, the important *C.ps*.-specific IFN-γ *in vitro* response of CD8^+^ T-cells (after re-stimulation with *C.ps*.-loaded WT BM derived DCs) failed in absence of non-myeloid C3 (Figure 5G). The *C.ps*.-induced increase of CD8^+^ effector-, effector memory-, and central memory T-cells also occurred only in both WT recipients (although without differences between the four *C.ps*. infected groups) suggesting that the homeostasis of these cells might also depend on systemic C3 (Figures 6A1–A4).

**Figure 7:**
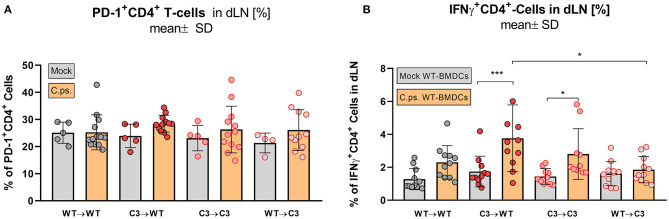
Formation of effector T-cells determined on day 20 *p.i*. BM chimeric mice (see also legend of Figure 1A) were infected with 750 IFU of *C.ps*.. On day 20 *p.i*., mice were sacrificed and dLN were isolated for flow cytometric analysis of T-lymphocytes and for re-stimulation experiments. PD-1 expression of CD4^+^ T-cells **(A)**, and percentage of IFN-γ^+^CD4^+^-T-cells after 16 h of re-stimulation with *C.ps*.-infected BM derived DCs were assessed **(B)**. Statistical analysis was performed by One-way ANOVA followed by Bonferroni post-test. Data were combined from 4 independent identically performed experiments. Statistical significance according to the calculated *p*-value: *p ≤ 0.0332; **p ≤ 0.0021; ***p ≤ 0.0002; ****p ≤ 0.0001.

In contrast, the effect of C3 on CD4^+^ T-cells was less striking (Figures 6B1–B4, 7). A significant increase of CD4^+^ effector-, effector memory- and central memory T-cells was only found in WT➔WT chimeric mice (Figure 7B). Moreover, in restimulation experiments, there was no detectable *C.ps*.-specific response of IFN-γ^+^ CD4^+^-cells in WT➔WT animals (Figure 7B). Although, CD4^+^ responses are often required for the induction and preservation of functional CD8^+^ responses to *Chlamydia* (57), they might occur at an earlier time point during *C.ps*. infection, as shown previously (46).

In our *C.ps*.-infected WT➔C3^−/−^ chimeric animals, the number of CD19^+^ B-cells in the dLN was also lower than that of infected WT➔WT mice, but similar to that of C3^−/−^➔C3^−/−^ animals. Moreover, there was no *C.ps*.-induced increase of B-cells due to myeloid cell-derived C3 alone (Figure 5E). The increase in *C.ps*.-specific IgG was also reduced in these mice (Figures 5E,I). In contrast, the specific IgM level was normal or even higher in C3^−/−^➔C3^−/−^ mice 20 day *p.i*. (Figure 5H).

The relatively mild infection with 750 IFU of *C.ps*. permitted prolonged observation including the period when adaptive immune responses are most important for defense. The majority of the results indicated a pivotal role of non-myeloid cell-derived complement in this experimental setting.

### Non-Myeloid Cell-Derived C3 Alone Is Sufficient and Crucial to Protect During Lung Infection With High Chlamydial Burden

**Figure 8:**
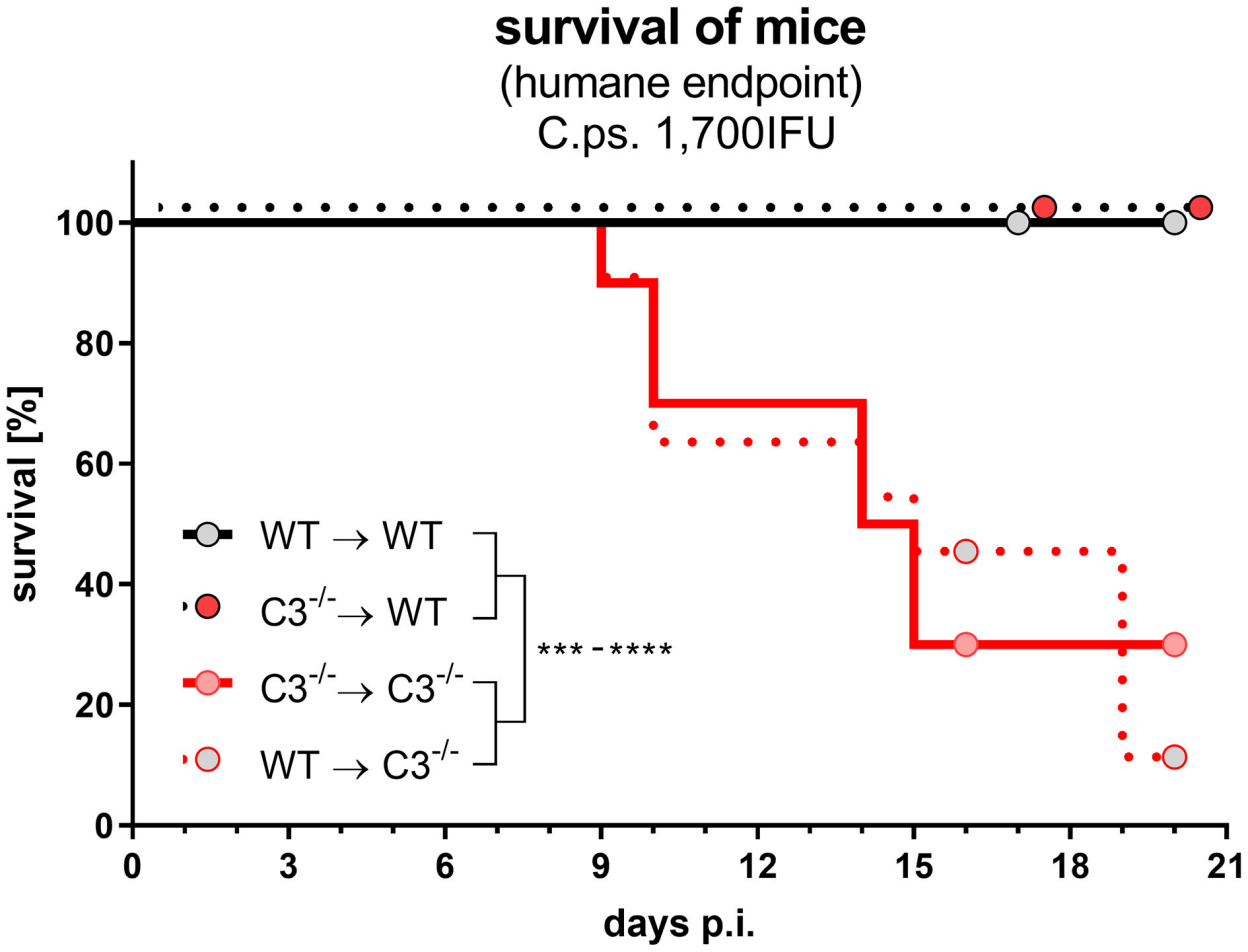
After lung infection with an increased number of *C. psittaci*, non-myeloid cell-derived C3 alone is essential for survival. A subset of the four different combinations of BM chimeric mice (see also legend of Figure 1A) was challenged with a higher dosage (1,700 IFU) of *C.ps*.. Under close monitoring the survival of these animals was assessed using humane endpoint defining criteria. The depicted results were obtained combining 4 independent identical experiments. Survival was analyzed by log-rank Mantel-cox test. Statistical significance according to the calculated *p*-value: *p ≤ 0.0332; **p ≤ 0.0021; ***p ≤ 0.0002; ****p ≤ 0.0001.

The different degree of the functional handicaps of the two mixed chimeras and thus, the much higher importance of non-myeloid compared to myeloid cell-derived C3 became even more apparent, when the *C.ps*. challenge experiment was performed with a higher IFU (1,700) per animal (Figure 8). All infected C3^−/−^➔WT (and WT➔WT) mice survived 20 days, whereas the majority of WT➔C3^−/−^ (and C3^−/−^➔C3^−/−^) chimeric mice reached already the humane endpoint between day 9 and 20.

Taken together, there was a drastic negative effect of the absence of non-myeloid-derived and a minor effect of the lack of myeloid-cell-derived complement on the development of the *C.ps*.-specific immune response and resulting protection/survival. Thus, obviously, C3 derived from non-myeloid cells plays the pivotal role.

## Discussion

In contrast to studies using genetically modified mice with T-cell receptor (TCR) specificity against an antigen that is artificially expressed on the pathogen (e.g., OT-I or OT-II mice), the work we present here describes a less pronounced, but most likely, more authentic antigen-directed immune responses in the context of a bacterial infection.

Our findings in a low IFU *C.ps*. infection on BM chimeric mice proved that both, C3 derived from myeloid- and non-myeloid sources, participate in the defense during chlamydial lung infection. The rather small effect of myeloid-cell-derived and most likely locally produced complement was surprising.

It seems highly unlikely that a reduction of the C3 levels in circulating plasma of ~ ≤ 5% could account for the observed differences between WT➔WT compared to C3^−/−^➔WT mice. It also appears questionable whether the remaining ≤ 5% of C3 in circulating plasma could be responsible for the observed differences between WT➔C3^−/−^ and C3^−/−^➔C3^−/−^ BM chimeric mice. Based on that reasoning and the intermediate phenotypes of the mixed chimeras in body weight and clinical score, it would appear plausible that complement locally produced by myeloid-derived cells and also locally consumed has at least some impact on the outcome of *C.ps*. infection.

Remarkably, by Western blot we could detect a small amount of C3 in the lungs of healthy WT➔C3^−/−^ but not of C3^−/−^➔C3^−/−^ mice. This suggests production of C3 in that organ by transferred C3^+/+^ myeloid-derived cells. It might be present in the lung tissue or its blood vessels. In any case, its biological effect in lung infections is obviously rather limited.

In one of the experiments, an increase in the infectious dose was performed to create more challenging conditions. Then, non-myeloid cell-derived, systemic C3 is notably more important than C3 produced by myeloid cells. This was illustrated by the fact that all C3^−/−^➔WT survived, whereas the majority of WT➔C3^−/−^ preliminary reached the humane endpoint.

In agreement with these findings, specific T- and B-cell responses, which are most likely essential for an effective defense from the second week of infection on, depended also mainly on non-myeloid cell-derived complement:

No *C.ps*.-induced increase of B-cells due to myeloid cell-derived C3 alone was observed. Furthermore, there was more IgM and less IgG in plasma of C3^−/−^ recipients on day 20 *p.i*.. That occurred most likely due to a delayed Ig class switch. This delay might be partially explained by the absence of C3b, because antibody class switch has been shown to depend on the interaction of the downstream degradation product C3d and CR2 (23).

Yet, a diminished *C.ps*.-induced rise of B-cells in the lung dLN also occurs in C3aR^−/−^ mice on day 9 of *C.ps*. infection (46), a finding which we could confirm for day 13 *p.i*. (data not shown). Thus, one noteworthy and important feature of C3a is also its participation in the regulation of the B-cell response during *C.ps*.-infection.

Others have shown that transferred B-cells from WT mice are able to functionally reconstitute C3 deficient mice for protection against viral infection. They drew the conclusion that production of complement by B-cells is sufficient to substitute complement deficiency (3). Intriguingly, in our experiments C3 derived from myeloid cells—which include the B-cells—did neither improve the impaired survival rate and protection after high IFU challenge, nor did it permit a regular humoral response against the bacteria in the low IFU lung infection model. Actually, the only obvious difference between WT➔C3^−/−^ and C3^−/−^➔C3^−/−^ BM chimeric mice was the reduced loss in body weight of the mixed chimera due to myeloid-cell-derived C3 during late stages after infection. It seems reasonable to conclude that (myeloid) C3 potentially derived from B-cells alone does not lead to a local environment in lymphoid organs, which enables a regular B-cell or T-cell response against *Chlamydia* in mice. Yet, we cannot exclude that the results might be different during infections with other pathogens.

After encountering chlamydial antigens by the TCR, cytotoxic CD8^+^ T-cells cooperate to induce death of *Chlamydia*-infected cells by lytic granule exocytosis and production of anti-microbial peptides, chemokines, and cytokines such as IFN-γ (57, 58). During chronic viral infection overstimulated CD8^+^ T-cells represent an exhausted functional restricted phenotype characterized by the expression of PD-1 (59, 60). Transcriptional profiling of *Chlamydia trachomatis* infection revealed a 2-fold increase in PD-1 ligand expression in plasmid containing more pathogenic strains as compared to plasmid deficient ones (61). Depletion of PD-1 ligand, augments memory CD8^+^ T-cell formation and CD8^+^ responses to *C. trachomatis* resulting in enhanced bacterial clearance (62). In addition, PD-1 blockage increases cytokine secretion of T-cells from *C. muridarum* immunized mice in co-culture with lung DCs (63).

In our study, non-myeloid-derived C3 was also essential for the *C.ps*.-dependent increase in the number of CD3^+^, CD4^+^, and CD8^+^ T-cells and the IFN-γ^+^ response of *C.ps*.-specific CD8^+^ T-cells. We observed higher PD-1 expression on CD8^+^ T-cells of both C3^−/−^ recipients, but not on their CD4^+^ T-cells. This strongly suggests that non-myeloid cell-derived C3 (or its cleavage product C3a) are required to sustain the functional status of CD8^+^ T-cells, and that CD8^+^ T-cell exhaustion occurs during *C.ps*. lung infection in its absence.

At this point, we cannot exclude the participation of other C3-dependent mechanisms leading to decreased growth rates or increased apoptosis of T-cells. However, at least in C3aR^−/−^ mice we could not find any significant differences in the percentage of Ki67^+^CD8^+^ T-cells, or 7-AAD and Annexin-V^+^-lymphocytes compared to *C.ps*.-infected WT mice (data not shown). Further studies will be required to decipher the underlying cellular and molecular mechanism.

This important role of complement we have revealed should also be considered in vaccine design for the development of optimal specific immune responses against chlamydiae. This might be achieved by the use of a conformationally-biased, response-selective agonists of complement components, e.g., of C5a: EP54, or EP67 (64), or fusion proteins of chlamydial antigens and the active C-terminal part of C3a as a molecular adjuvant (65), or by addition of C3b deposition on chlamydial antigens to serve as molecular adjuvant (23), or the use of complement activating adjuvants such as algammulin (66).

Of course, there may be several additional critical, complement-related factors potentially influencing the outcome of *C.ps*. lung infection. However, analysis of the kinetics of complement-dependent protection and the impact on DC migration are beyond the scope of this work and are being addressed in a closely related, parallel study.

The recent advances in the complement field have revolutionized the understanding of its role in immune responses during infections. In this context, a prominent role of locally produced complement has been proposed (6, 11). Several supporting experiments were conducted in *in vitro* co-culture systems with mouse (14) or isolated and stimulated human immune-cells (4). In contrast, our data are derived from a more authentic and physiologic model of pneumonia caused by intracellular *C.ps*. Our results prove the major importance of non-myeloid-derived, circulating/systemic C3 in protection and adaptive T- and B-cell responses in mice. Myeloid-derived, local C3 seems to play here only a minor, mainly fine-tuning or modulating role.

Further studies are required to clarify how far our finding concerning the different roles and importance of complement derived from the two sources might also apply to infections of mice or humans with other intracellular pathogens, or extracellular pathogens causing prolonged infections.

## Data Availability Statement

The raw data supporting the conclusions of this article will be made available by the authors, without undue reservation.

## Ethics Statement

The animal study was reviewed and approved by Lower Saxony State Office for Consumer Protection and Food Safety (LAVES), permit: 33.12-42502-04-18/2751.

## Author Contributions

MK: investigation (performed mouse model and analyses of the obtained samples, in particular flow cytometry), formal analysis (statistics), methodology (established new methods in the lab, e.g., for DC migration), visualization (original graphs), participated in conceptualization and planning, wrote most of the initial and part of the final draft of the manuscript. CR: performed experiments with the samples obtained in the mouse model, e.g., ELISAs, RL: performed mouse model and part of the analyses of the samples obtained there. SG: performed blinded histological scoring of mouse lung tissue. CL: participated in the mouse model. AK: conceptualization, funding acquisition, supervision, visualization (optimization of graphs), wrote parts of the initial draft and optimized the manuscript for submission. All authors contributed to the article and approved the submitted version.

## Conflict of Interest

The authors declare that the research was conducted in the absence of any commercial or financial relationships that could be construed as a potential conflict of interest.
