# Supplementary material for: Complement and Chlamydia psittaci: Non-Myeloid-Derived C3 Predominantly Induces Protective Adaptive Immune Responses in Mouse Lung Infection
Source: Front Immunol. 2021 Mar 4;12:626627. doi: 10.3389/fimmu.2021.626627 (PMC7969653; doi:10.3389/fimmu.2021.626627)
Supplement: Supplementary file 1 [file Data_Sheet_2.pdf]

Sup. Table 1.: Clinical scoring (pneumoniae and post-irradiation)

Parameter	Clinical Observation	Points
Piloerection	Normal coated fur	0
	Partial ruffed fur	1
	Slightly ruffed fur	2
	Ruffed fur	3
Body posture	Normal posture	0
	Slightly hunched posture	1
	Hunched posture	2
Locomotion	Spontaneous movement, movement after cage opening or provocation of sleeping animals, normal behavior	0
	Movement only after provocation	1
	No movement – **humane endpoint**	2
Agility	Normal and fast movements	0
	Slow and / or sluggish	1
Breathing (Pneumoniae only)	Unaffected	0
	Tachypnea	1
	Tachypnea with abdominal effort while breathing, gasping	2
Reaction to tactile stimulation	Normal attentiveness, escape reflex at approach	0
	Attentiveness slightly affected, reduced reaction to external stimuli	1
	Somnolent – **humane endpoint**	2
Ocular or nostril discharge	None	0
	Presence of ocular and / or nostril discharge (→ treatment with eye ointment)	1
	Conjunctivitis with thick ocular discharge (→ treatment with eye ointment)	2
Dehydration (exsiccosis)	Regular skin turgor	0
	Reduced skin turgor, delayed spread of skin fold (→ mashed and soaked wet food on cage floor)	1
	Poor skin turgor, no spread of skin fold – **humane endpoint**	2
Body weight loss	0-3%	0
	3-10%	1
	10-20%	2
	>20%	3
Sum (Pneumoniae)	Sum of clinical score ≥ 14 – **humane endpoint**	Σ19
	Control frequency: daily	
	measures to increase animal well-being	
Sum (post-irradiation)	Sum of clinical score ≥ 12 – **humane endpoint** (breathing not taken into account)	Σ17
	Control frequency:	
	day 0-5: daily	
	day 6-14: three times if clinical score ≤ 5	
	week 3-6: once if clinical score ≤ 5	

Sup. Table 2: Scoring system to evaluate the pathology of deparaffinized lung sections

Criteria	Score	Pathology
number of inflammatory cells	0	none, number of macrophages normal
	1	low number of inflammatory cells
	2	high number of inflammatory cells
degree of tissue bleeding	0	none
	1	low number of erythrocytes in alveoli
	2	high number of erythrocytes in alveoli
presence of oedema	0	none
	1	moderate
	2	extensive
affected areas	0	none
	1	0-10%
	2	10-20%
	3	25-50%
	4	50-75%
	5	>75%
peribronchial infiltrate	0	none
	1	low
	2	moderate
	3	evident
	4	extensive
	5	severe
luminal exudation	0	none
	1	low
	2	moderate
	3	evident
	4	extensive
	5	severe
overall pathology	0	none
	1	low, 10%
	2	moderate, 10-25%
	3	evident, 25-50%
	4	extensive, 50-75%
	5	severe >75%

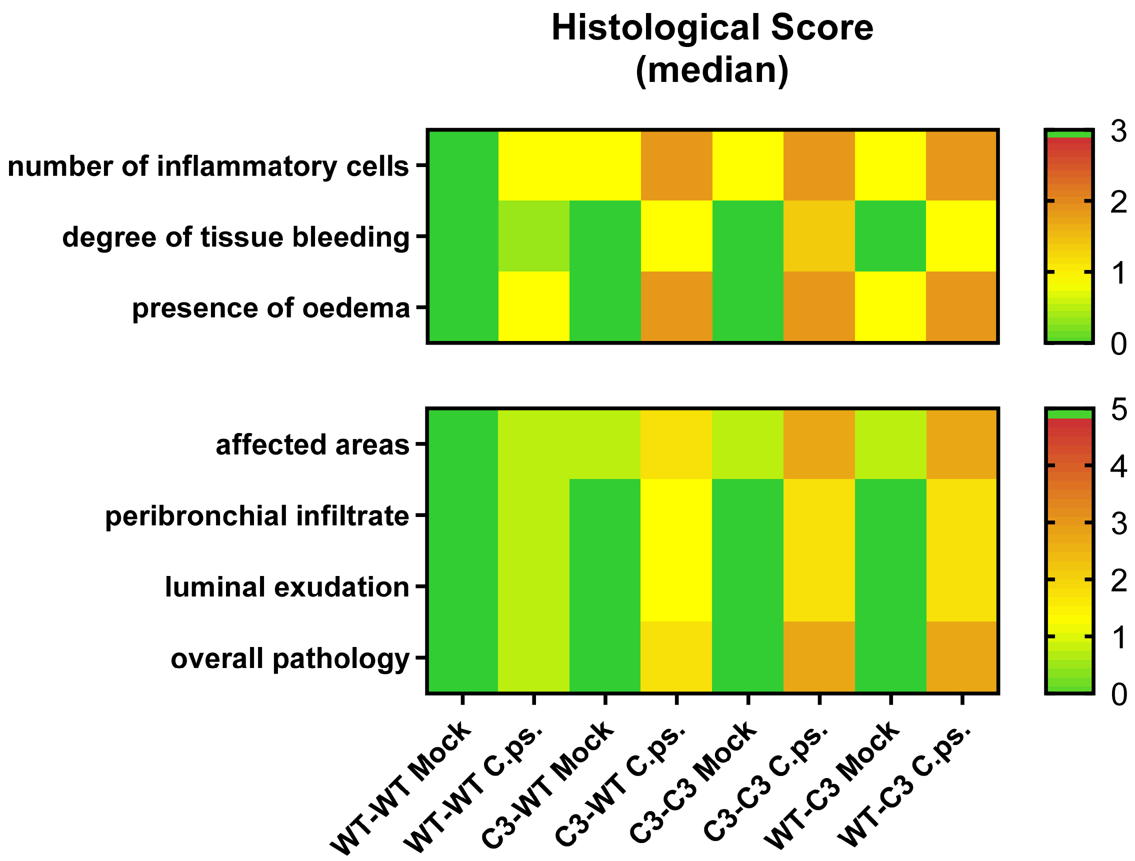

Sup. Fig.1.: Histological score of each parameter to evaluate the pathology of deparaffinized lung sections referring to the overall score represented in Fig. 3.

Sup. Table 3: Histological score of each parameter to evaluate the pathology of deparaffinized lung sections referring to the overall score represented in Fig. 3.

median of score	number of inflammatory cells	degree of tissue bleeding	presence of oedema	affected areas	peribronchial infiltrate	luminal exudation	overall pathology	sum
**WT-WT Mock**	0	0	0	0	0	0	0	**0**
**WT-WT C.ps.**	1	0,5	1	1	1	1	1	**7**
**C3-WT Mock**	1	0	0	1	0	0	0	**2**
**C3-WT C.ps.**	2	1	2	2	1,5	1,5	2	**12,5**
**C3-C3 Mock**	1	0	0	1	0	0	0	**2**
**C3-C3 C.ps.**	2	1,5	2	3	2	2	3	**15,5**
**WT-C3 Mock**	1	0	1	1	0	0	0	**3**
**WT-C3 C.ps.**	2	1	2	3	2	2	3	**15**
